# Dissecting the roles of β-arrestin2 and GSK-3 signaling in 5-HT1BR-mediated perseverative behavior and prepulse inhibition deficits in mice

**DOI:** 10.1371/journal.pone.0211239

**Published:** 2019-02-05

**Authors:** Summer L. Thompson, Stephanie C. Dulawa

**Affiliations:** 1 Department of Psychiatry, University of California San Diego, La Jolla, California, United States of America; 2 Committee on Neurobiology, University of Chicago, Chicago, Illinois, United States of America; Biomedical Sciences Research Center Alexander Fleming, GREECE

## Abstract

Serotonin-1B receptors (5-HT1BRs) modulate perseverative behaviors and prepulse inhibition (PPI) in humans and mice. These inhibitory G-protein-coupled receptors signal through a canonical G-protein-coupled pathway that is modulated by GSK-3β, and a noncanonical pathway mediated by the adaptor protein β-arrestin2 (Arrb2). Given the development of biased ligands that differentially affect canonical versus noncanonical signaling, we examined which signaling pathway mediates 5-HT1BR agonist-induced locomotor perseveration and PPI deficits, behavioral phenotypes observed in both obsessive-compulsive disorder (OCD) and autism spectrum disorder (ASD). To assess the role of canonical 5-HT1BR signaling, mice received acute pretreatment with a GSK-3 inhibitor (SB216763 or AR-A014418) and acute treatment with the 5-HT1A/1B receptor agonist RU24969 prior to assessing perseverative locomotor behavior in the open field, and PPI. To determine the role of noncanonical 5-HT1BR signaling, *Arrb2* wild-type (WT), heterozygous (HT), and knockout (KO) mice received acute RU24969 treatment prior to behavioral testing. GSK-3 inhibition increased locomotor perseveration overall, and also failed to influence the RU24969-induced perseverative locomotor pattern in the open field. Yet, GSK-3 inhibition modestly reduced RU24969-induced PPI deficits. On the other hand, *Arrb2* HT and KO mice showed reduced locomotion and no changes in perseveration overall, in addition to modest reductions in RU24969-induced locomotion and PPI deficits. In conclusion, our data do not support use of either GSK-3 inhibitors or β-arrestin2 inhibition in treatment of perseverative behaviors.

## Introduction

Serotonin-1B receptors (5-HT1BRs), previously termed 5-HT1Dβ in humans [1], modulate perseverative behavior and prepulse inhibition (PPI) in humans [2–5] and mice [6–9]. Perseverative behavior refers to the inappropriate and inflexible repetition of a behavior, while PPI is a form of plasticity of the startle reflex that is thought to quantify sensorimotor gating, the ability to filter out extraneous sensory, cognitive, and motor information [10]. Perseverative behavior and deficient PPI are features of several neuropsychiatric disorders, including obsessive-compulsive disorder (OCD) and autism spectrum disorders (ASD) [11]. Some evidence suggests that perseverative behavior and PPI levels may be correlated [12,13]. Currently, chronic treatment with serotonin reuptake inhibitors (SRIs) provides the only pharmacological monotherapy for treating perseverative symptoms in OCD and ASD [14–16]. Thus, novel treatments for these disorders represent a major unmet need.

Acute treatment with the 5-HT1A/1B receptor agonist RU24969 induces PPI deficits and a highly perseverative pattern of locomotion in the open field in rodents [6–8]. Similarly, 5-HT1BR agonists exacerbate OCD symptoms [2,3,5] and growth hormone responses associated with baseline repetitive behaviors in ASD [17]. The behavioral effects of RU24969 are mediated through 5-HT1BRs, but not 5-HT1ARs, since pretreatment with a 5-HT1BR antagonist, but not a 5-HT1AR antagonist, blocks these effects [8]. The RU24969-induced perseverative locomotor pattern is characterized by hyperactivity, reduced vertical rearing, and a rigid circling path, which can be quantified using the spatial scaling exponent *d* (spatial *d*). Spatial *d* quantifies the smoothness of the animal’s path, where highly circumscribed paths have high spatial *d* and paths with few directional changes have low spatial *d*, which is characteristic of RU24969-induced locomotor perseveration [18]. RU24969-induced locomotor perseveration and PPI deficits can be prevented by four weeks of pretreatment with SRIs, but not norepinephrine reuptake inhibitors [6–8], paralleling treatment effects in OCD and ASD [14,19].

5-HT1BRs are G-protein-coupled receptors (GPCRs) that bind G_iα2_ to inhibit adenylyl cyclase and cAMP production [20,21]. 5-HT1BRs signal through a canonical, G-protein-coupled pathway, and at least one noncanonical G-protein-independent pathway that involves beta-arrestin2 (β-arrestin2) [22]. Canonical 5-HT1BR signaling is mediated by direct interaction with glycogen synthase-3 beta (GSK-3β), a constitutively active serine/threonine protein kinase that stabilizes the complex between 5-HT1BRs and G_iα2_, and is integral for downstream G-protein-mediated signaling [22,23]. This interaction is specific, as GSK-3β does not modulate signaling of 5-HT1ARs; furthermore, the other GSK-3 isoform, GSK-3α, does not modulate 5-HT1BR signaling [22]. The intracellular adaptor protein, β-arrestin2, interacts with 5-HT1BRs in an activity-dependent manner and mediates noncanonical signaling [23,24]. Whether canonical or noncanonical signaling at 5-HT1BRs induces perseverative behavior and PPI deficits is unknown. Given the recent identification of biased ligands that differentially stimulate canonical versus β-arrestin2-mediated 5-HT1BR signaling [25], it is of great interest to identify the pathway mediating 5-HT1BR-induced perseverative behavior and PPI deficits to develop novel therapeutics.

Here, we investigated the role of canonical versus noncanonical 5-HT1BR signaling in RU24969-induced perseverative locomotion and PPI deficits. To determine the contribution of canonical 5-HT1BR signaling, mice received pretreatment with a GSK-3 inhibitor, and then RU24969-induced effects on locomotion and PPI were assessed. To assess the contribution of noncanonical 5-HT1BR signaling, *Arrb2* wild-type (WT), heterozygous (HT), and knockout (KO) mice were assessed for RU24969-induced effects on locomotion and PPI testing.

## Materials and methods

### Animals

Experiment-naïve female, 8-week old, Balb/cJ mice (Experiments 1–4) were purchased from Jackson Laboratories (Bar Harbor, ME) and acclimated to the animal facility for 1 week prior to undergoing experimental procedures. Balb/cJ females were used for Experiments 1–4 for consistency with our prior studies that validated this model for the study of OCD-like behaviors [7–9]. For Experiment 5, male and female *Arrb2* WT, HT, and KO mice on a C57BL/6J background, aged 7 to 11 weeks, were bred in-house through heterozygous crossings from mice purchased from Jackson Laboratories (Stock #: 023852; Bar Harbor, ME). Male mice were included in Experiment 5 because male mice were recently shown to exhibit OCD-like behaviors that are responsive to fluoxetine [26], much like females. C57BL/6J mice were used in Experiment 5 because this strain was also recently validated in the RU24969-induced model [6] and this design allowed for reduced waste of animals (such as additional animals needed for backcross breeding or waste of animals by excluding all male offspring). However, due to these design differences, results of Experiment 5 were not directly compared to those of Experiments 1–4. Estrous cycle was not assessed because to our knowledge there is no evidence that the estrous cycle plays a role in RU24969-induced behavioral changes in female mice and the interaction of hormones with 5-HT1BR signaling was not a primary interest in this investigation. Animals were housed in a climate-controlled room maintained on a 12 hour:12 hour light:dark cycle. All testing was performed during the light cycle. Mice had ad libitum access to standard chow and water. All procedures were conducted in accordance with the National Institutes of Health laboratory animal care guidelines and with the Institutional Animal Care and Use Committee of the University of Chicago, Protocol #71671 (Experiments 2, 4, and 5) or University of California San Diego, Protocol #S15266 (Experiments 1 and 3). All efforts were made to minimize suffering, and animals were sacrificed at the end of behavioral testing using standard carbon dioxide euthanasia followed by a secondary method.

### Drugs

All drugs were administered via intraperitoneal injection. 5-HT1A/B agonist RU24969 (Tocris Bioscience, Bristol, UK) was dissolved in saline (0.9% NaCl) and administered at 0 or 3 mg/kg (Experiments 1 and 3), 0 or 10 mg/kg (Experiments 2 and 4) or 0, 3, or 10 mg/kg (Experiment 5) at 5 ml/kg injection volume. Doses were selected based on previous studies demonstrating OCD-like behavior in this dose range [6–9] and to achieve a high and a more moderate dose [27]. The GSK-3 inhibitor SB216763 (Tocris Bioscience, Bristol, UK) was dissolved in 4% DMSO/15% Tween-80 in saline and injected at 20 ml/kg injection volume. SB216763 was administered at 0, 5, or 10 mg/kg based on previous studies demonstrating effects of SB216763 on relevant behavioral measures and the ability of SB216763 to block behavioral effects of other pharmacological compounds in this dose range [28–30]. GSK-3 inhibitor AR-A014418 (Tocris Bioscience, Bristol, UK) was dissolved in 4% DMSO/15% Tween-80 in saline and injected at 20 ml/kg injection volume. AR-A014418 was administered at 0, 10, or 20 mg/kg based on previous studies [31,32]. SB216763 and AR-A014418 were used because they have high selectivity for GSK-3 inhibition, modulate 5-HT1BR signaling, and cross the blood-brain barrier [23,33–35].

### Behavioral testing

#### Open field

The open field test was performed as described previously [7]. Briefly, mice were placed in a corner of the open field and activity was monitored for 20 minutes. All measures were automatically generated by the Versamax program (Accuscan, Columbus, OH) with the exception of the spatial scaling exponent “spatial *d*,” which was calculated using Python (Python Software Foundation, Beaverton, OR), NightOwl (custom software), and BMDP software. Spatial *d* describes the geometric pattern of activity, with higher values indicating a more circumscribed path and lower values indicating a straighter path characteristic of locomotor perseveration [18].

#### Prepulse inhibition

PPI refers to the reduction in startle magnitude that occurs when an abrupt startling stimulus is preceded by a brief prepulse [10]. PPI was assessed as described previously [7]. Briefly, mice were placed in startle chambers (San Diego Instruments, San Diego, CA) and amplitude of the startle response was measured for 65 ms in response to five types of trials lasting 40 ms each for a total of 62 trials: pulse alone (40 ms broadband burst at 120 dB), three different prepulse inhibition trials (20 ms prepulses 3, 6, or 12 dB above background followed 100 ms later by a 120 dB pulse), or no stimulus, in which only background noise was presented. The test session was 20 minutes long, comprised of a 5 min acclimation period, a block of six startle trials (Block 1), two blocks of 25 intermixed trial types (Blocks 2 and 3), then a final block of six startle trials (Block 4). Percent PPI was calculated as follows: 100*(startle response—prepulse-inhibited startle response)/startle response. For all experiments assessing PPI, PPI testing occurred directly following open field testing.

### Experiments

#### Experiment 1

To assess the contribution of canonical GPCR signaling to RU24969-induced behaviors, mice were pseudorandomly assigned to receive one of three pretreatments (0, 5, or 10 mg/kg SB216763) and one of two treatments (0 or 3 mg/kg RU24969; n = 12-14/group) in a between-subject design. SB216763 was administered 30 minutes prior to open field testing based on timing of peak behavioral effects in previous studies [30]. RU24969 was administered 5 minutes prior to open field testing to be consistent with findings from our previous studies [6–9]. Animals underwent PPI testing directly following open field testing.

#### Experiment 2

To further investigate the effect of GSK-3 inhibition on RU24969-induced phenotypes, mice were pseudorandomly assigned to receive one of three pretreatments (0, 5, or 10 mg/kg SB216763) and one of two treatments (0 or 10 mg/kg RU24969; n = 14/group) in a between-subject design. Treatment timing and behavioral assessment were as in Experiment 1.

#### Experiment 3

Effects of SB216763 on RU24969-induced behaviors in the open field were next assessed at an earlier time point. Mice and treatments were as in Experiment 1 (n = 13-14/group). SB216763 was administered 60 minutes prior to open field testing. RU24969 was administered 35 minutes prior to open field testing.

#### Experiment 4

To corroborate findings using SB216763, alternative GSK-3 inhibitor AR-A014418 was tested for effects on RU24969-induced behaviors. Mice were pseudorandomly assigned to receive one of three pretreatments (0, 10, or 20 mg/kg AR-A014418) and one of two treatments (0 or 10 mg/kg RU24969; n = 14/group) in a between subject design. Treatment timing was the same as Experiments 1–2 based on behavioral effects in previous studies [31]. Behavioral testing was as in Experiment 1.

#### Experiment 5

To test the contribution of the noncanonical β-arrestin2 signaling pathway to RU24969-induced behaviors, male and female *Arrb2* WT, HT, and KO mice (n = 13-15/sex/genotype) all received 0, 3, and 10 mg/kg RU24969 in a counterbalanced fashion, in a within-subject design. Treatment timing and behavioral testing were as in Experiment 1.

### Statistical analysis

For all experiments, dependent measures were analyzed using repeated measures analysis of variance (ANOVA). Significant interactions were resolved by assessing simple main effects in ANOVAs. Significant simple main effects were resolved by tests for simple contrasts: post hoc ANOVAs for within-subject variables or Student Newman-Keuls tests for between-subject variables. P-values from post hoc ANOVAs were assessed for significance using the Bonferroni correction. Alpha was set at 0.05. Open field measures were analyzed with bin (4 × 5 minutes) as a repeated measure. PPI was analyzed with block and prepulse intensity as repeated measures, and startle was also analyzed with block as a repeated measure. These repeated measures (bin, block, prepulse intensity) were included in order to assess effects over the course of testing and are only stated in the text and represented in figures for which interactions with factors of interest (genotype, pretreatment, treatment) occurred. In Experiment 5, RU24969 treatment was analyzed as an additional repeated measure and sex was assessed as an independent factor, but sex effects were not reported because sex did not have any three-way interactions with the primary factors of interest: genotype and treatment. Furthermore, a secondary analysis was performed, in which only mice with a matched genotype control for total distance traveled within the saline condition were included, where matched mice had activity levels disparities of less than 100 cm or 5% of total distance traveled. Then, the same analyses were performed. Effect sizes were assessed using Cohen’s d. Pearson’s correlations and simple regressions were assessed.

## Results

### GSK-3 inhibition does not affect RU24969-induced open field phenotypes

**Fig 1:**
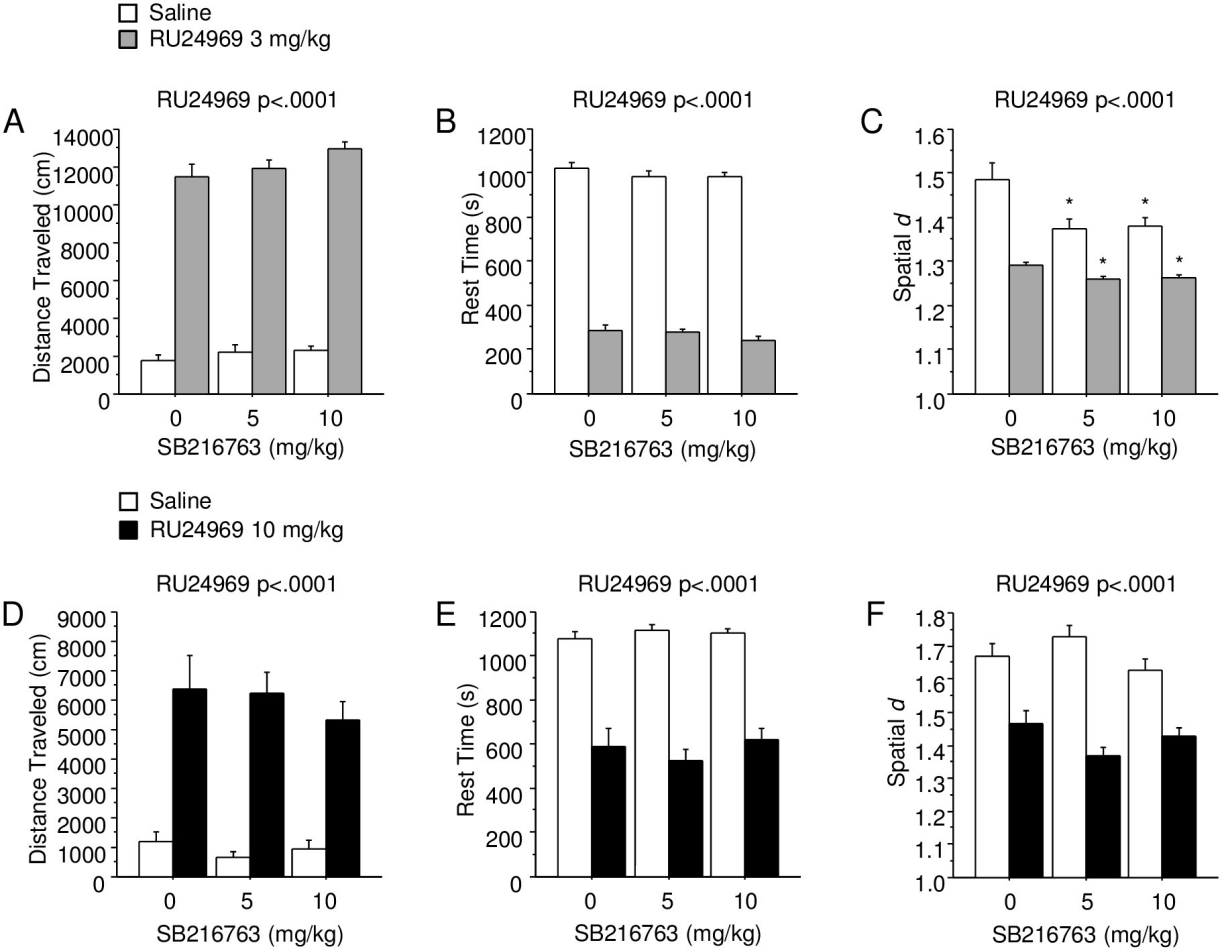
GSK-3 inhibition did not alter RU24969-induced changes in locomotor behavior in the open field test. A—C show distance traveled (A), time spent resting (B), and spatial d (C) for Experiment 3 (n = 13-14/group), in which mice were administered GSK-3 inhibitor SB216763 60 minutes prior to behavioral testing and 0 (white bars) or 3 mg/kg (gray bars) RU24969 35 minutes prior to behavioral testing. D—F show distance traveled (D), time spent resting (E), and spatial d (F) for Experiment 2 (n = 14/group), in which mice were administered GSK-3 inhibitor SB216763 30 minutes prior to behavioral testing and 0 (white bars) or 10 mg/kg (black bars) RU24969 5 minutes prior to behavioral testing. Results expressed as mean ± SEM. *Significant difference from vehicle within pretreatment or treatment groups. SEM: standard error of the mean.

**Fig 2:**
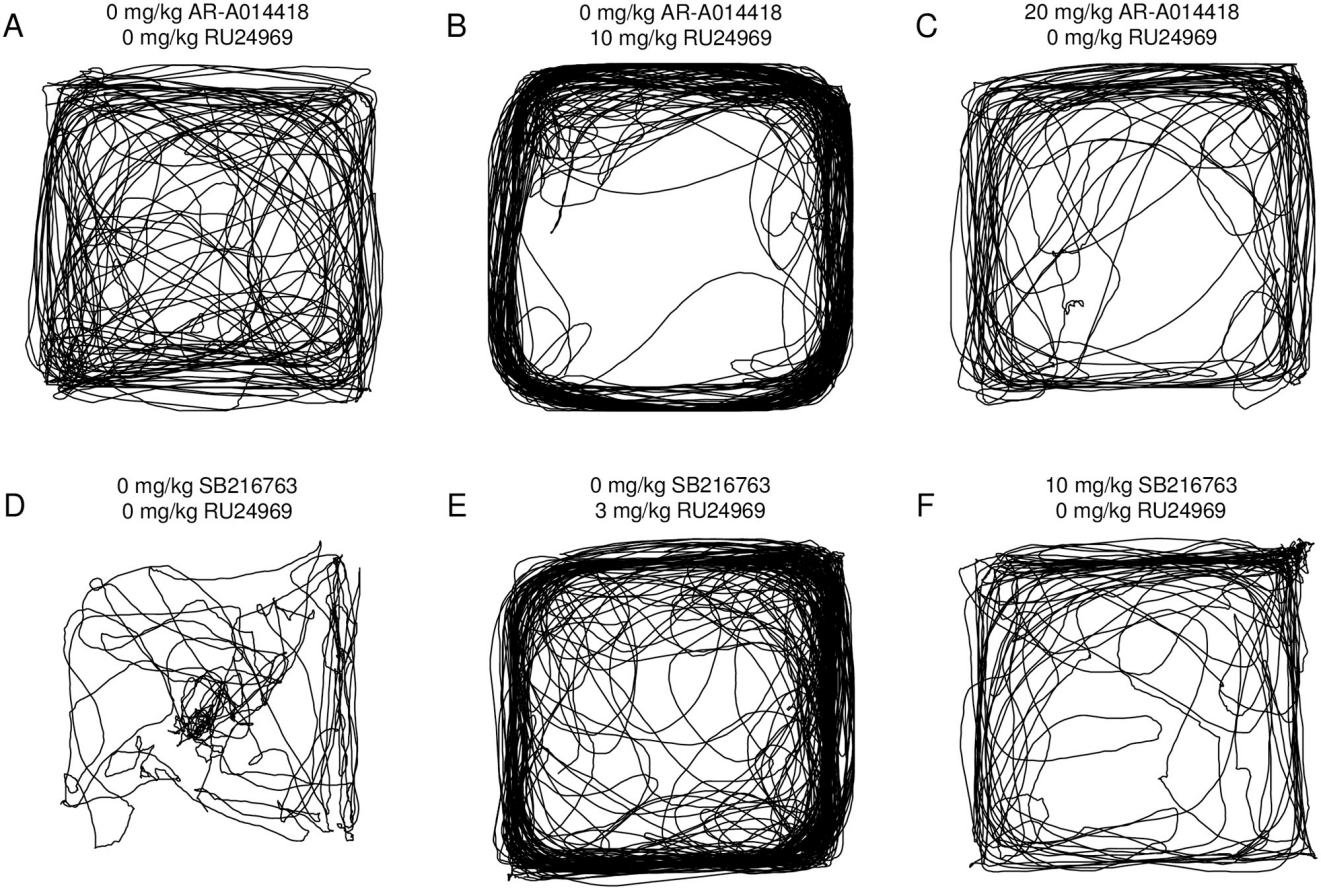
Both RU24969 and GSK-3 inhibitors reduce spatial *d*. Example traces of open field paths from mice in Experiment 4 (A-C) and Experiment 3 (D-F). RU24969-treated (B, E) and GSK-3 inhibitor-treated (C, F) mice have a similar pattern of activity characteristic of low spatial *d*, in contrast to mice treated only with vehicle (A, D).

For Experiment 1, no effects of SB216763 pretreatment were found on 3 mg/kg RU24969-induced locomotor perseveration in the open field (S1 Fig). Thus, effects of SB216763 on behavior were assessed at a later time point in Experiment 3. In the open field, RU24969 treatment increased distance traveled across pretreatment groups at the low dose, 3 mg/kg (F_(1,76)_ = 846.10; p < .0001; Experiment 3; Fig 1A), and at the high dose, 10 mg/kg (F_(1,78)_ = 96.83; p < .0001; Experiment 2; Fig 1D). For 3 mg/kg RU24969, SB216763 pretreatment had a trend for an effect on total distance traveled (F_(2,76)_ = 2.79; p = .07), but had no interaction with RU24969 treatment. SB216763 pretreatment had no effect on distance traveled for Experiment 2. Similarly, RU24969 reduced time spent resting across pretreatment groups for low-dose (F_(1,76)_ = 1613.13; p < .0001; Fig 1B) and high-dose treatment (F_(1,78)_ = 158.58; p < .0001; Fig 1E), whereas SB216763 had no effect on rest time in either experiment. The 3 mg/kg dose of RU24969 decreased spatial *d* across SB216763 groups (F_(1,74)_ = 23.32; p < .0001; Fig 1C), as did 10 mg/kg (F_(1,78)_ = 24.26; p < .0001; Fig 1F). There was no main effect of SB216763 pretreatment on spatial *d* in either experiment. However, planned comparisons were performed, which revealed that both 5 mg/kg and 10 mg/kg SB216763 reduced spatial *d* in both the saline and 3 mg/kg RU24969-treated groups (Figs 1c and 2f). In contrast, SB216763 had no effect on spatial *d* in Experiment 2, in which mice received high dose RU24969 treatment 5 minutes prior to open field testing. The absence of GSK-3 inhibitor interaction with RU24969 (high-dose) to affect open field measures was confirmed using a second GSK-3 inhibitor, AR-A014418 (S2 Fig; S1 Supplemental Results).

### GSK-3 inhibition mitigates RU24969-induced PPI deficits

**Fig 3:**
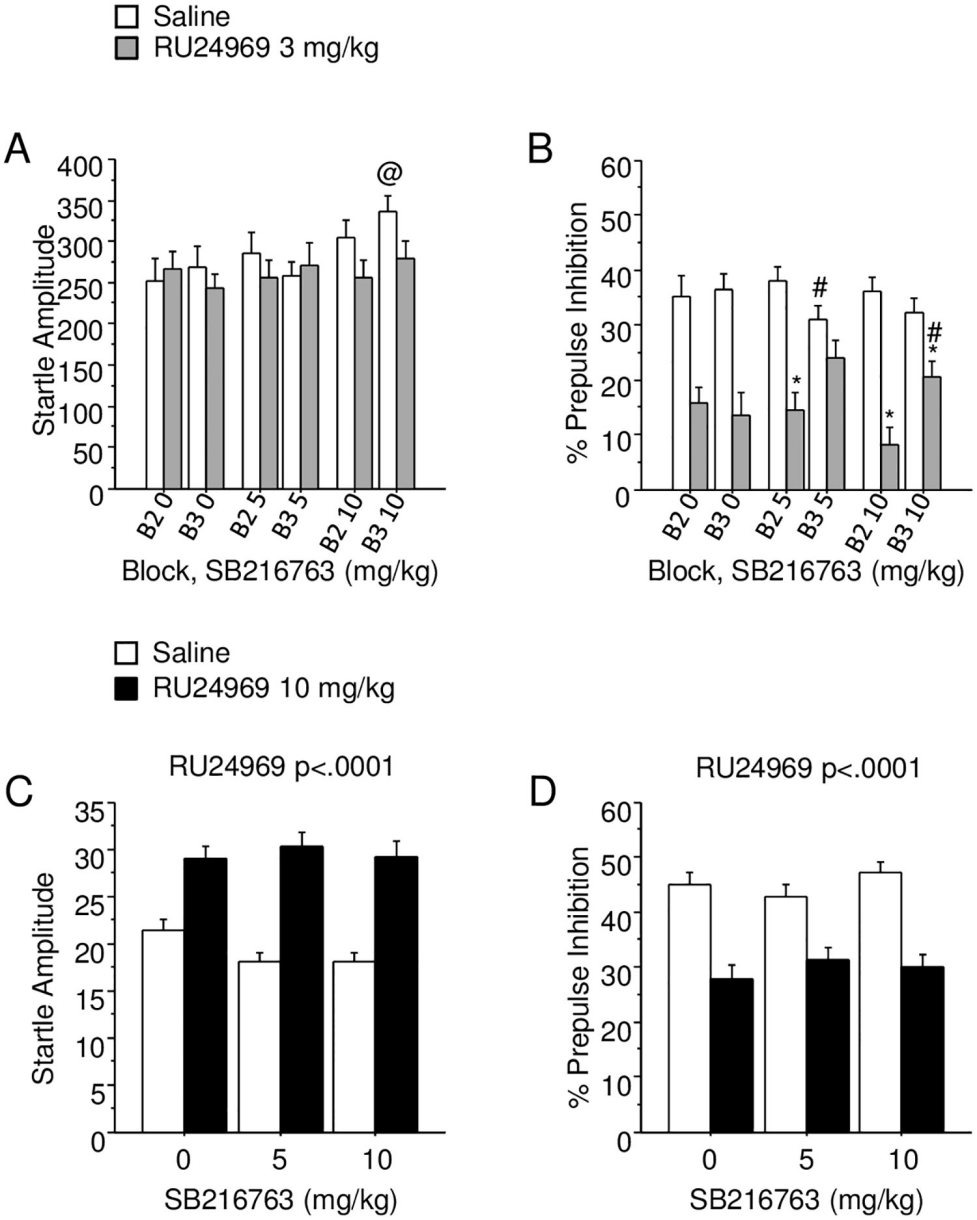
GSK-3 inhibition mitigated low dose, but not high dose, RU24969-induced changes in prepulse inhibition. A—B show startle amplitude (A) and percent PPI (B) for Experiment 1 (n = 12-14/group), in which mice were pretreated with GSK-3 inhibitor SB216763 30 minutes prior to initiating behavioral testing (50–55 minutes prior to PPI testing) and 0 (white bars) or 3 mg/kg (gray bars) RU24969 5 minutes prior to initiating behavioral testing (25–30 minutes prior to PPI testing). C—D show startle amplitude (C) and percent PPI (D) for Experiment 2, in which mice were pretreated with SB216763 30 minutes prior to behavioral testing and 0 (white bars) or 10 mg/kg (black bars) RU24969 5 minutes prior to initiating behavioral testing. Results expressed as mean ± SEM. *Significant difference from saline treated group within pretreatment and block. #Significant difference from block 2 within pretreatment and treatment group. @Significant difference from both other pretreatment groups within treatment group within block. PPI: prepulse inhibition SEM: standard error of the mean.

In Experiment 1, there was a three-way interaction among RU24969, SB216763 and Block for startle amplitude (F_(2,72)_ = 4.53; p < .05; Fig 3A). Post hoc analysis revealed that within saline-treated mice, 10 mg/kg SB216763 increased startle amplitude over 0 or 5 mg/kg SB216763-pretreated groups in Block 3 but not Block 2. SB216763 pretreatment mitigated low-dose RU24969-induced PPI deficits in the second half of testing, as revealed by a three-way interaction among SB216763, RU24969, and Block (F_(2,70)_ = 3.89; p < .05; Fig 3B). Post hoc analyses revealed that RU24969 reduced PPI across blocks in saline-pretreated mice (F_(1,22)_ = 18.19; p < .0005), whereas RU24969 reduced PPI in Block 2 but not Block 3 for 5 mg/kg SB216763-pretreated mice. In addition, for 5 mg/kg SB216763-pretreated, saline-treated mice, PPI was reduced in Block 3 relative to Block 2 (F_(1,12)_ = 14.98; p < .005). RU24969 reduced PPI in both blocks for 10 mg/kg SB216763-pretreated mice, but the 3 mg/kg RU24969 group had increased PPI in Block 3 relative to Block 2 (F_(1,11)_ = 9.79; p < .01).

In Experiment 2, RU24969 increased startle amplitude overall (F_(1,78)_ = 57.50; p < .0001; Fig 3C), whereas SB216763 had no effect. High-dose RU24969 treatment decreased PPI overall (F_(1,78)_ = 25.07; p < .0001; Fig 3D), whereas SB216763 had no effect. The lack of interaction between GSK-3 inhibition and high-dose RU24969 treatment for PPI measures was confirmed using another GSK-3 inhibitor, AR-A014418 (S2 Fig; S1 Supplemental Results).

### *Arrb2* expression affects RU24969-induced open field phenotypes

**Fig 4:**
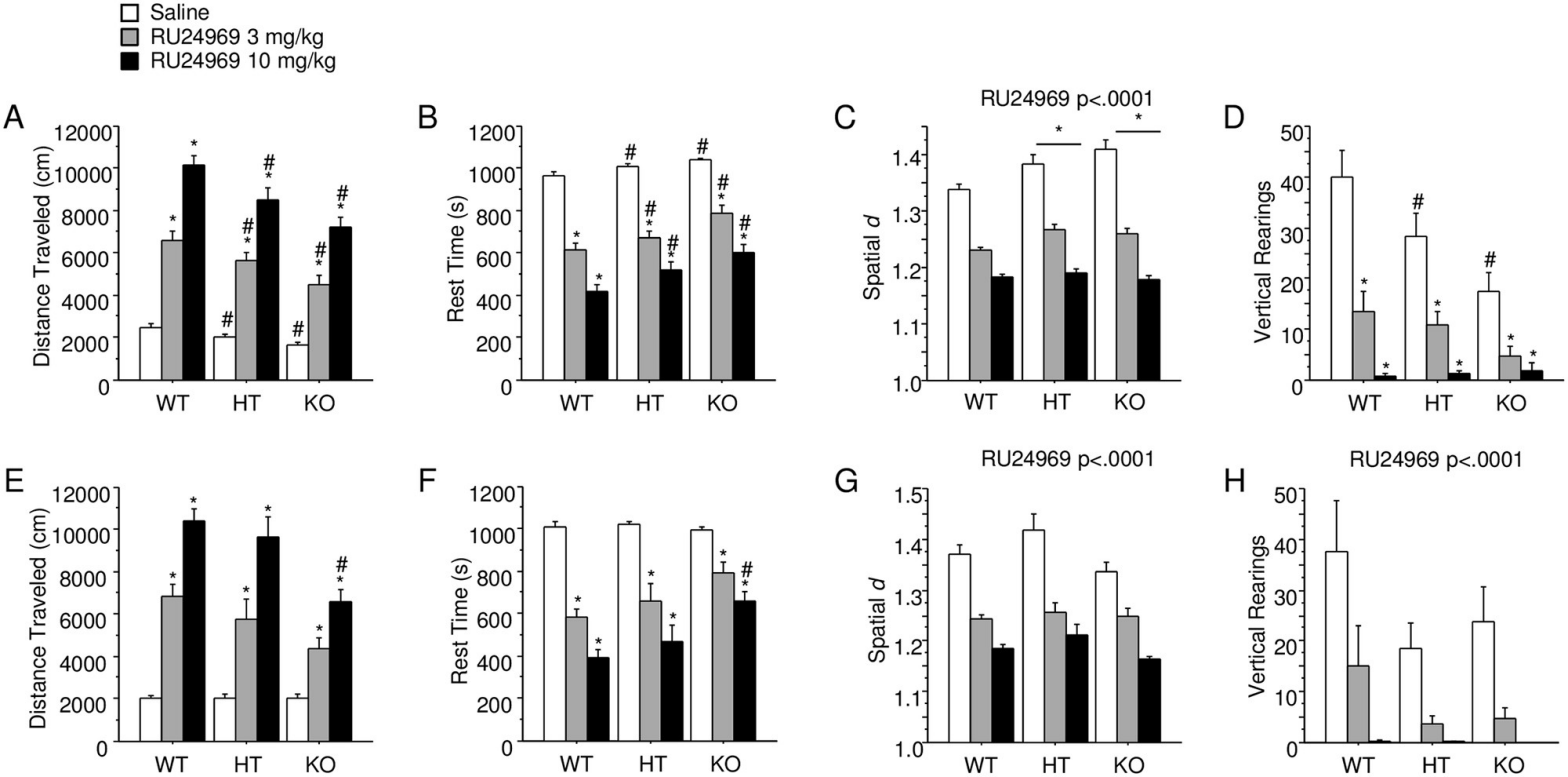
*Arrb2* genotype modulates RU24969-induced locomotor effects in the open field test. A—D show raw locomotor data: distance traveled (A), time spent resting (B), spatial *d* (C), and instances of vertical rearing (D) for all mice from Experiment 5 (n = 13-15/sex/genotype), in which *Arrb2* WT, HT, and KO, male and female mice were administered 0 (white bars), 3 (gray bars), or 10 mg/kg (black bars) RU24969 5 minutes prior to initiating behavioral testing. E—H show data corrected for baseline locomotor activity levels in the secondary analysis (n = 4-6/sex/genotype): distance traveled (E), rest time (F), spatial *d* (G), and instances of vertical rearing (H). Results expressed as mean ± SEM. *Significant difference from saline treatment group within genotype. #Significant difference from WT within treatment condition. *Significant difference from WT collapsed across treatment groups. SEM: standard error of the mean WT: wild-type HT: heterozygous KO: knockout.

A genotype by treatment interaction was found for distance traveled (F_(4,156)_ = 3.09; p < .05; Fig 4A). Post hoc tests revealed that both 3 mg/kg and 10 mg/kg RU24969 increased distance traveled for WT (3 mg/kg: F_(1,28)_ = 89.52; p < .0001; 10 mg/kg: F_(1,28)_ = 241.40; p < .0001), HT (3 mg/kg: F_(1,27)_ = 87.13; p < .0001; 10 mg/kg: F_(1,27)_ = 141.17; p < .0001) and KO mice (3 mg/kg: F_(1,26)_ = 52.48; p < .0001; 10 mg/kg: F_(1,27)_ = 127.49; p < .0001). Post hoc tests also revealed that *Arrb2* HT and KO mice traveled less distance than WT mice within each treatment condition, including saline. To assess the relationship between saline- and RU24969-induced activity levels, within-subject correlation and regression analyses were performed between saline and RU24969 conditions. These tests revealed no relationship between saline and RU24969-induced activity levels within any genotype (S1 Supplemental Results).

A trend for a genotype by treatment interaction was found for rest time (F_(4,156)_ = 1.99; p = .099; Fig 4B). Post hoc tests revealed that both 3 mg/kg and 10 mg/kg RU24969 decreased rest time within each genotype and that HT and KO mice rested more than WT mice within each RU24969 dose. To assess the relationship between saline- and RU24969-induced rest times, within-subject correlation and regression analyses were performed between saline and RU24969 conditions. This assessment identified no relationship between saline- and RU24969-induced rest times within each genotype (S1 Supplemental Results).

There was a main effect of genotype on spatial *d* (F_(2,75)_ = 3.45; p < .05; Fig 4C). Post hoc tests revealed that both HT and KO mice had higher spatial *d* than WT mice. There was a main effect of RU24969 on spatial *d* (F_(2,150)_ = 150.30; p < .0001). Post hoc tests revealed that both 3 mg/kg and 10 mg/kg RU24969 reduced spatial *d*.

A genotype by RU24969 treatment interaction was observed for vertical activity (F_(4,156)_ = 4.73; p < .005) (Fig 4D). Post hoc tests revealed that both 3 mg/kg and 10 mg/kg RU24969 reduced the number of vertical rearings within each genotype. Post hoc tests also revealed that HT and KO mice had fewer vertical rearings than WT mice within the saline condition. None of the assessed measures showed a three-way interaction among sex, genotype, and treatment.

Because of the reduced distance traveled in *Arrb2* HT and KO mice even in the saline condition, consistent with previous reports of hypoactivity in *Arrb2* KO mice [36–39], a secondary analysis was performed in order to assess the relationship between genotype and treatment in the absence of a baseline effect of genotype on distance traveled. Mice were matched by genotype for total distance traveled in the saline condition (n = 4-6/sex/genotype). Mice without matches were excluded. This matching procedure resulted in an elimination of the effect of genotype on distance traveled in the open field in the saline condition (F_(2,29)_ = .003; p = .99). The behavior of this subset of mice was then reanalyzed for all effects. A genotype by treatment interaction was found for distance traveled in the matched set of mice (F_(4,50)_ = 3.28; p < .05; Fig 4E). Post hoc tests revealed that both 3 mg/kg and 10 mg/kg RU24969 still increased distance traveled for WT (3 mg/kg: F_(1,10)_ = 69.02; p < .0001; 10 mg/kg: F_(1,10)_ = 162.15; p < .0001), HT (3 mg/kg: F_(1,8)_ = 15.19; p < .005; 10 mg/kg: F_(1,9)_ = 58.76; p < .0001) and KO mice (3 mg/kg: F_(1,10)_ = 21.332; p = .001; 10 mg/kg: F_(1,10)_ = 47.84; p < .0001). Post hoc tests also revealed that *Arrb2* KO mice traveled a shorter total distance than WT or HT mice, within the 10 mg/kg RU24969 condition, but not within the 3 mg/kg condition after Bonferroni correction (F_(2,28)_ = 3.54; p = .04).

Using the same set of mice matched for distance traveled in the saline condition, rest time was reassessed. As expected, there was no longer an effect of genotype on time spent resting in the saline condition (F_(2,29)_ = .47; p = .63). A genotype by treatment interaction was observed for time spent resting (F_(4,50)_ = 2.85; p < .05; Fig 4F). Post hoc tests revealed that 3 mg/kg and 10 mg/kg RU24969 decreased rest time in WT (3 mg/kg: F_(1,10)_ = 96.12; p < .0001; 10 mg/kg: F_(1,10)_ = 161.66; p < .0001), HT (3 mg/kg: F_(1,8)_ = 16.83; p < .005; 10 mg/kg: F_(1,9)_ = 42.41; p = .0001) and KO mice (3 mg/kg: F_(1,10)_ = 15.00; p < .005; 10 mg/kg: F_(1,10)_ = 40.78; p < .0001). Post hoc tests also revealed that KO mice rested more than WT or HT mice in the 10 mg/kg RU24969 condition, but not after Bonferroni correction in the 3 mg/kg RU24969 condition (F_(2,28)_ = 3.71; p = .04).

Using the same set of mice as above, spatial *d* was reanalyzed. The previous main effect of genotype lost significance with this subset of mice (F_(2,24)_ = 2.09; p = .15). A main effect of RU24969 treatment was observed (F_(2,48)_ = 65.60; p < .0001; Fig 4G). Post hoc tests revealed that both 3 mg/kg RU24969 (F_(1,29)_ = 61.00; p < .0001) and 10 mg/kg RU24969 decreased spatial *d* (F_(1,30)_ = 100.78; p < .0001).

Using the saline activity-matched cohort, vertical activity was reassessed. There was no longer an effect of genotype on vertical rearing in the saline-treated condition (F_(2,29)_ = 1.518; p = .24). A main effect of RU24969 treatment was observed (F_(2,50)_ = 23.41; p < .0001; Fig 4H). Post hoc tests revealed that both 3 mg/kg RU24969 (F_(1,30)_ = 23.87; p < .0001) and 10 mg/kg RU24969 decreased vertical activity (F_(1,31)_ = 33.41; p < .0001). Overall, These activity-matched results confirmed that *Arrb2* KO mice traveled a shorter distance than WT or HT mice receiving 10 mg/kg RU24969 (Fig 4E).

### *Arrb2* expression affects RU24969-induced PPI deficits

**Fig 5:**
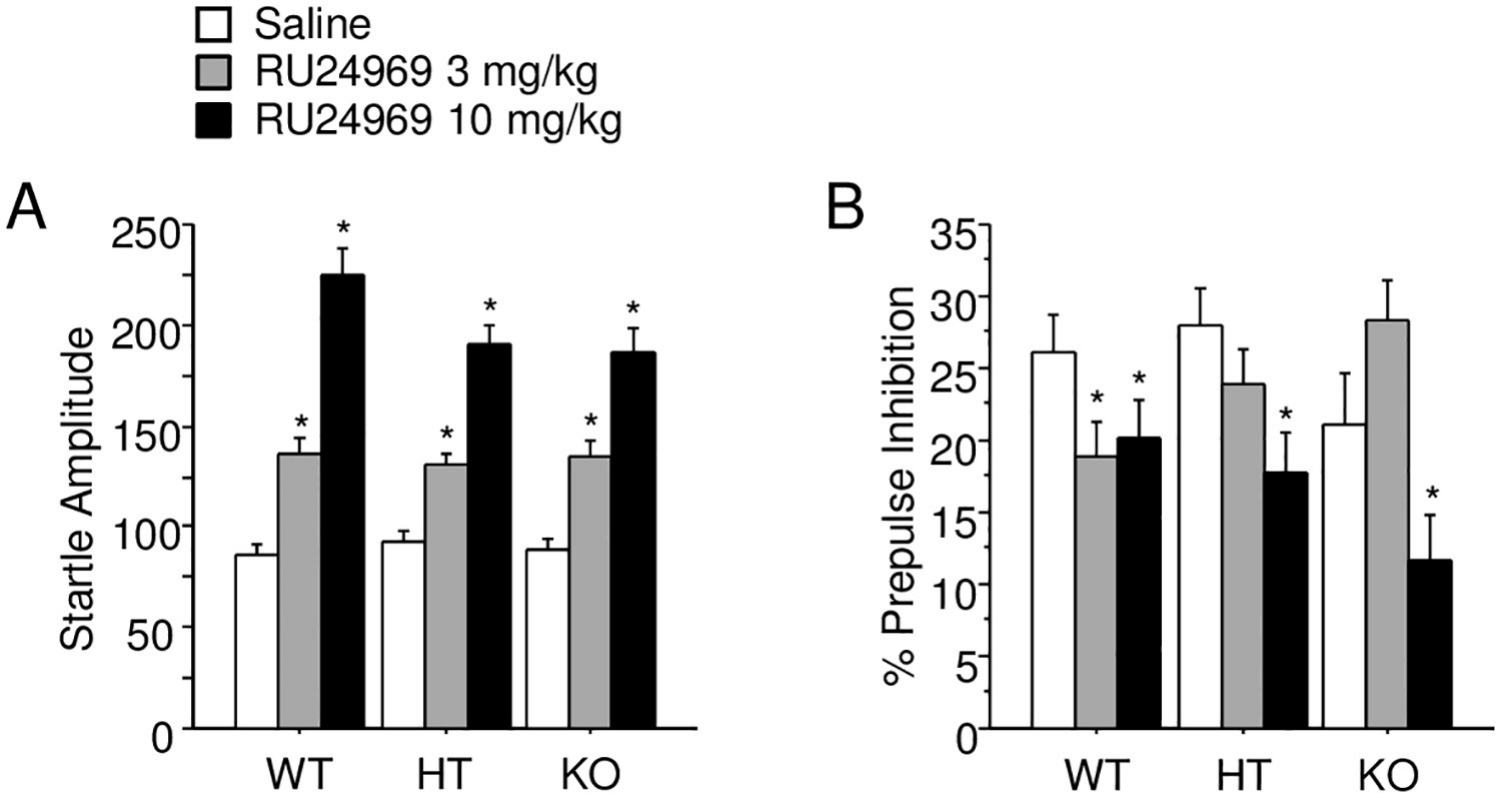
Reduced *Arrb2* expression prevents 3 mg/kg RU24969-induced PPI deficits. A—B show startle amplitude (A) and PPI (B) for Experiment 5 (n = 13-15/sex/genotype), in which *Arrb2* WT, HT, and KO, male and female mice were administered 0 (white bars), 3 (gray bars), or 10 mg/kg (black bars) RU24969 5 minutes prior to initiating behavioral testing. Results expressed as mean ± SEM. *Significant difference from saline treatment group within genotype. PPI: prepulse inhibition SEM: standard error of the mean WT: wild-type HT: heterozygous KO: knockout.

A trend was found for a genotype by treatment interaction for startle amplitude (F_(4,164)_ = 2.08; p = .09; Fig 5A). Post hoc tests revealed that 3 mg/kg and 10 mg/kg RU24969 increased startle for WT (3 mg/kg: F_(1,29)_ = 29.74; p < .0001; 10 mg/kg: F_(1,29)_ = 86.58; p < .0001), HT (3 mg/kg: F_(1,29)_ = 22.83; p < .0001; 10 mg/kg: F_(1,29)_ = 98.13; p < .0001), and KO mice (3 mg/kg F_(1,27)_ = 20.57; p = .0001; 10 mg/kg: F_(1,27)_ = 57.56; p < .0001) whereas *Arrb2* genotype had no effect at any dose of RU24969.

A genotype by treatment interaction was observed for PPI (F_(4,164)_ = 2.66; p < .05; Fig 5B). Post hoc tests revealed that 10 mg/kg RU24969 reduced PPI in KO mice (F_(1,27)_ = 56.59; p < .0001), whereas simple main effects of RU24969 treatment missed significance in WT (F_(2,58)_ = 2.73; p = .07) and HT mice (F_(2,58)_ = 3.11; p = .05). Since RU24969 has consistently been reported to reduce PPI [7,8], planned comparisons were performed for the effects of each RU24969 dose within genotype. Planned comparisons revealed that 3 mg/kg RU24969 reduced PPI in WT, but not HT or KO, mice. Planned comparisons also revealed that 10 mg/kg RU24969 reduced PPI within each genotype.

## Discussion

Our present findings show that both GSK-3 inhibition and *Arrb2* knockout modestly reduce 5-HT1BR-induced PPI deficits, but do not diminish 5-HT1BR-induced locomotor perseveration. Specifically, neither GSK-3 inhibition nor *Arrb2* knockout altered RU24969-induced reductions in spatial *d* or rearing behavior. *Arrb2* knockout was found to reduce 5-HT1BR-induced hyperactivity, which reflects the amount but not the perseverative quality of locomotion, while GSK-3 inhibition had no effect on RU24969-induced hyperactivity. Thus, both canonical and noncanonical 5-HT1BR signaling plays a modest role in RU24969-induced PPI deficits, but not 5-HT1BR-induced perseverative behavior. Overall, our findings do not support a role for 5-HT1BR antagonists biased toward canonical or β-arrestin2-mediated signaling pathways as therapeutics for perseverative or compulsive behaviors.

The only effect of GSK-3 inhibition on behavior in the open field was to reduce spatial *d*. SB216763 reduced spatial *d* in mice receiving saline or 3 mg/kg RU24969 (S1 Fig). This effect was observed sixty minutes, but not thirty minutes, after injection of SB216763. Furthermore, AR-A014418 also reduced spatial *d* in mice receiving saline or 10 mg/kg RU24969 (S2 Fig). No interaction of GSK-3 inhibition and RU24969 treatment was identified for spatial *d*, indicating that GSK-3 modulates spatial *d* independent of 5-HT1BR signaling. Thus, GSK-3 inhibition increased locomotor stereotypy in the open field, much like RU24969 (Fig 2). On the other hand, GSK-3 inhibition did not alter RU24969-induced increases in locomotion. RU24969 induced robust hyperactivity concomitant with decreases in rest time (Fig 1 and S1 and S2 Figs), paralleling our previous work [6–8]. Our findings are in line with previous evidence that pharmacological or genetic inhibition of GSK-3 failed to mitigate hyperactivity induced by anpirtoline, a 5-HT1BR agonist with a similar pharmacological profile [23,40]. Mice lacking GSK-3β only in serotonin neurons, snGSK-3β KO mice, were reported to lack anpirtoline-induced reductions in vertical rearing [40]. However, we were unable to assess this effect due to low baseline levels of rearing in the Balb/cJ strain [41] relative to C57BL/6J mice [6]. Overall, GSK-3 inhibition only appears to increase locomotor perseveration via a 5-HT1BR-independent mechanism in the open field.

We found that GSK-3 inhibition mitigated RU24969-induced PPI deficits (Fig 3B). SB216763 partially blocked the PPI deficit induced by 3 mg/kg RU24969 in the third testing block, likely indicating a delayed onset of the interaction between RU24969 and SB216763. The attenuation of RU24969-induced PPI deficits by SB216763 were not due to any confounding effects on startle magnitude (Fig 3A), since the effects of GSK-3 inhibition on startle amplitude were dissociable from those on PPI. Furthermore, GSK-3 inhibition by SB216763 reduced 3 mg/kg, but not 10 mg/kg, RU24969-induced PPI deficits (Fig 3). Most likely, the lack of effect of AR-A014418 on RU24969-induced PPI deficits was due to the use of the 10 mg/kg dose of RU24969 in this study (S2 Fig). These modest effects of GSK-3 inhibition on RU24969-induced PPI deficits suggest a minor role for GSK-3 signaling in 5-HT1BR-mediated PPI deficits. The absence of effects of SB216763 alone on PPI conflicts with previous reports, indicating that experimental factors such as route of administration, testing time point, strain, or sex may influence these effects [29,42]. For example, GSK-3 activity was previously shown to positively correlate with PPI across a panel of mouse strains [43]. In sum, GSK-3 inhibition attenuates the disruption of PPI induced by 3 mg/kg RU24969.

*Arrb2* genotype affected baseline locomotor activity levels (Fig 4A and 4B), with modest reductions in locomotion observed in HT and KO mice, consistent with previous reports [36–39]. We therefore normalized activity levels by creating matched groups across genotypes for baseline locomotion; this dataset was used for all analyses (Fig 4E–4H). *Arrb2* KO mice showed diminished hyperlocomotion and increased rest time in response to 10 mg/kg RU24969 compared to WT mice in the open field (Fig 4E and 4F). In addition, effect sizes for RU24969 treatment on distance traveled were substantially larger in WT than HT or KO mice (S1 Supplemental Results). Our findings parallel previous reports that *Arrb2* knockout prevents anpirtoline-induced hyperactivity [23]. Overall, *Arrb2* KO mice show diminished RU24969-induced hyperlocomotion, regardless of baseline activity levels.

*Arrb2* KO did not affect measures of perseverative locomotion including spatial *d* and vertical rearing in the open field. Although *Arrb2* HT and KO mice showed increases in spatial *d* (Fig 4C), this effect was lost in the activity-matched subset analysis (Fig 4G). Baseline spatial *d* in WT mice was notably lower in Experiment 5 than Experiments 1–4, consistent with previous spatial *d* levels found in C57BL/6J [6] versus Balb/cJ mice [7,8]. Furthermore, no effect of *Arrb2* genotype on vertical rearing was found in the activity-matched subset analysis (Fig 4H). This finding is consistent with a previous report of no effect of *Arrb2* genotype on rearing [36], although the effect may have simply lost power due to the reduced sample size combined with the high variability in rearing behavior in these mice. RU24969 robustly decreased rearing overall, as previously reported (Fig 4D)[6].

As we observed with GSK-3 inhibition, *Arrb2* HT and KO mice also showed attenuation of RU24969-induced PPI deficits. However, comparison of these findings must be made with caution due to use of different genetic background strains. In contrast to WT mice, *Arrb2* HT and KO mice did not exhibit PPI deficits following treatment with 3 mg/kg RU24969. However, 10 mg/kg RU24969 treatment reduced PPI in all genotypes (Fig 5). These effects on PPI were not artifacts of changes in startle magnitude, since RU24969 increased startle comparably in all genotypes. *Arrb2* KO might have reduced the effects of 3, but not 10 mg/kg RU24969 because the high dose induces more robust effects across behavioral domains [6], and thus subtle ameliorative effects may be occluded at this dose. PPI levels in Experiment 5 were notably lower than in Experiments 1–4, likely due to the low PPI levels characteristic of the C57BL/6J strain [44]. Thus, reduction in β-arrestin2-mediated signaling appears to reduce RU24969-mediated PPI deficits at lower, but not higher, doses.

Some aspects of the RU24969-induced behavioral syndrome were not modulated by GSK-3 inhibition or *Arrb2* KO. For example, direct measures of RU24969-induced locomotor stereotypy including reduced spatial *d* and reduced rearing, were not altered by inhibiting either pathway. One possible explanation for these findings is that another noncanonical signaling pathway mediates the effects of 5-HT1BR activation. For example, G-protein coupled-receptor kinases (GRKs) classically phosphorylate GPCRs to recruit arrestins for desensitization or endocytosis. However, emerging evidence indicates that GRKs also modulate activity of key intracellular signaling molecules, such as ERK and Akt [45]. Another possibility is that the effects of blocking β-arrestin2 signaling were obscured by developmental compensations in the *Arrb2* KO mice.

The present studies have several limitations. First, the *Arrb2* KO mice were on a C57BL/6J genetic background, while Balb/cJ mice were used for GSK-3 inhibition studies. Second, the *Arrb2* KO mice may have compensatory developmental changes that could obscure the effects of lacking β-arrestin2. Indeed, such compensatory changes can cause effects that are opposite to those of acute drug action at the gene product [46], highlighting the need for development of selective β-arrestin2 inhibitors. However, despite numerous reports of altered pharmacological responses and baseline hypoactivity in these mice [36–39], *Arrb2* KO mice are grossly normal, including metrics such as body weight, food intake, and basal body temperature [47–49]. Furthermore, the recently developed floxed β-arrestin2 mouse [50], and viral approaches for altering β-arrestin2 expression provide future tools for identifying the role of β-arrestin2 signaling. Third, RU24969 activates both 5-HT1A and 5-HT1BRs although the behaviors we investigated here are mediated by 5-HT1BRs, rather than 5-HT1A receptor activation [6–9]. Fourth, the GSK-3 inhibitors used here, SB216763 and AR-A014418, do not distinguish between the alpha and beta isoforms. Yet, GSK-3α does not interact with 5-HT1BRs, in contrast to GSK-3β [23]. Nevertheless, general effects of GSK-3 inhibition might involve off-target effects of GSK-3β interacting with substrates other than 5-HT1BRs, or effects of GSK-3α. In addition, while we selected doses and timing of GSK-3 inhibitor administration based on positive findings in the literature [30–32], we cannot rule out the possibility that a different combination of doses and timing of administration may yield different results. Finally, we did not assess estrous cycle in female mice in these studies. However, to our knowledge there is no evidence of estrous cycle influencing RU24969-induced behavioral effects. Furthermore, elegant studies have shown that female rodents do not consistently have increased phenotypic variability than males, suggesting that this measurement should not be required in order to use female subjects [51,52]. This is consistent with our results in Experiment 5, in which we did not identify systematic differences in coefficient of variation between sexes (data not shown). In sum, our design allowed us to perform a preliminary assessment of the role of downstream signaling pathways in a clinically relevant model of OCD-like behavior.

Pharmacological 5-HT1BR challenge exacerbates OCD symptoms [2,3,5] and induces a larger growth hormone response in ASD that correlates with baseline levels of repetitive behaviors [17]. Furthermore, the 5-HT1A/1BR antagonist pindolol potentiates the effects of chronic SRI treatment in treatment-resistant OCD patients [53–55]. These findings suggest that 5-HT1BR antagonists might ameliorate perseverative behavior in these disorders. However, our findings do not support a role for GSK-3 inhibitors or β-arrestin2 inhibition in the treatment of perseverative behaviors. Neither manipulation attenuated RU24969-induced locomotor perseveration as quantified by spatial *d*, or reductions in rearing. In fact, GSK-3 inhibition worsened locomotor perseveration overall. On the other hand, our findings suggest that GSK-3 inhibitors or β-arrestin2 inhibition might attenuate PPI deficits in disorders such as OCD and ASD, although the clinical relevance of improving PPI alone remains unclear. Lastly, the attenuation of PPI deficits induced by these agents was very modest in the present studies.

In conclusion, both GSK-3- and β-arrestin2-dependent intracellular signaling pathways mediate aspects of 5-HT1BR-induced PPI deficits, but not locomotor perseveration in mice. Additionally, GSK-3 inhibitors were found to increase locomotor perseveration overall, and thus might be detrimental to patients with neuropsychiatric disorders characterized by perseverative behaviors or compulsions. Future studies should examine the effects of selective β-arrestin2 inhibitors once they become available.

## Supporting information

S1 Fig GSK-3 inhibition does not alter 3 mg/kg RU24969-induced locomotor effects in the open field test. A—C show distance traveled (A), time spent resting (B), and spatial d (C) for Experiment 1 (n = 12-14/group), in which GSK-3 inhibitor SB216763 was administered 30 minutes prior to initiating behavioral testing and 0 (white bars) or 3 mg/kg (gray bars) RU24969 was administered 5 minutes prior to initiating behavioral testing. Results expressed as mean ± SEM. SEM: standard error of the mean.
(TIF)

S2 Fig GSK-3 inhibition with a second GSK-3 inhibitor did not affect high dose RU24969-induced behavior in the open field or prepulse inhibition. A—E show effects of AR-A014418 and RU24969 on open field measures in Experiment 4 (n = 14/group), in which AR-A014418 was administered 30 minutes prior to initiation of behavioral testing and 0 (white bars) or 10 mg/kg (black bars) RU24969 was administered 5 minutes prior to initiation of behavioral testing: total distance traveled (A), time spent resting (B), or spatial d (C). D—E show effects of AR- A014418 and RU24969 on startle amplitude (D) and percent prepulse inhibition (E). Results expressed as mean ± SEM. *Significantly different from vehicle pretreatment across treatment groups. SEM: standard error of the mean.
(TIF)

S1 Supplemental Results Supplemental results for Experiments 1 and 4. Associated with S1 and S2 Figs.
(PDF)

S1 Data Experiment 1 data. Excel spreadsheet including raw data from Experiment 1 open field and PPI tests.
(XLS)

S2 Data Experiment 2 data. Excel spreadsheet including raw data from Experiment 2 open field and PPI tests.
(XLS)

S3 Data Experiment 3 data. Excel spreadsheet including raw data from Experiment 3 open field test.
(XLS)

S4 Data Experiment 4 data. Excel spreadsheet including raw data from Experiment 4 open field and PPI tests.
(XLS)

S5 Data Experiment 5 data. Excel spreadsheet including raw data from Experiment 5 open field and PPI tests.
(XLS)
