# Supplementary figures and images for: Dissecting the roles of β-arrestin2 and GSK-3 signaling in 5-HT1BR-mediated perseverative behavior and prepulse inhibition deficits in mice
Source: PLoS One. 2019 Feb 5;14(2):e0211239. doi: 10.1371/journal.pone.0211239 (PMC6363181; doi:10.1371/journal.pone.0211239)

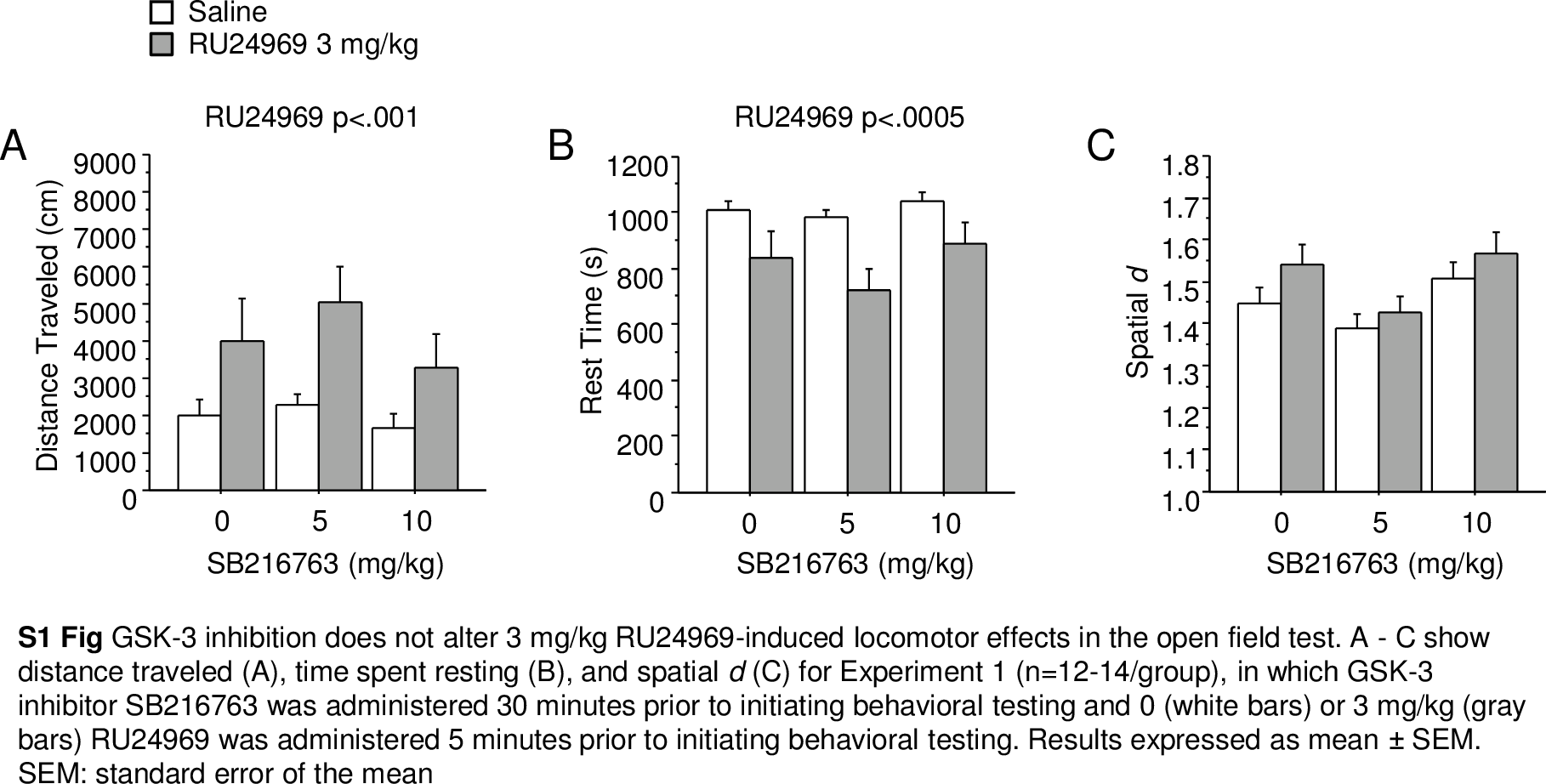

Supplement: S1 Fig — A—C show distance traveled (A), time spent resting (B), and spatial d (C) for Experiment 1 (n = 12-14/group), in which GSK-3 inhibitor SB216763 was administered 30 minutes prior to initiating behavioral testing and 0 (white bars) or 3 mg/kg (gray bars) RU24969 was administered 5 minutes prior to initiating behavioral testing. Results expressed as mean ± SEM. SEM: standard error of the mean. (TIF) [file pone.0211239.s001.tif]

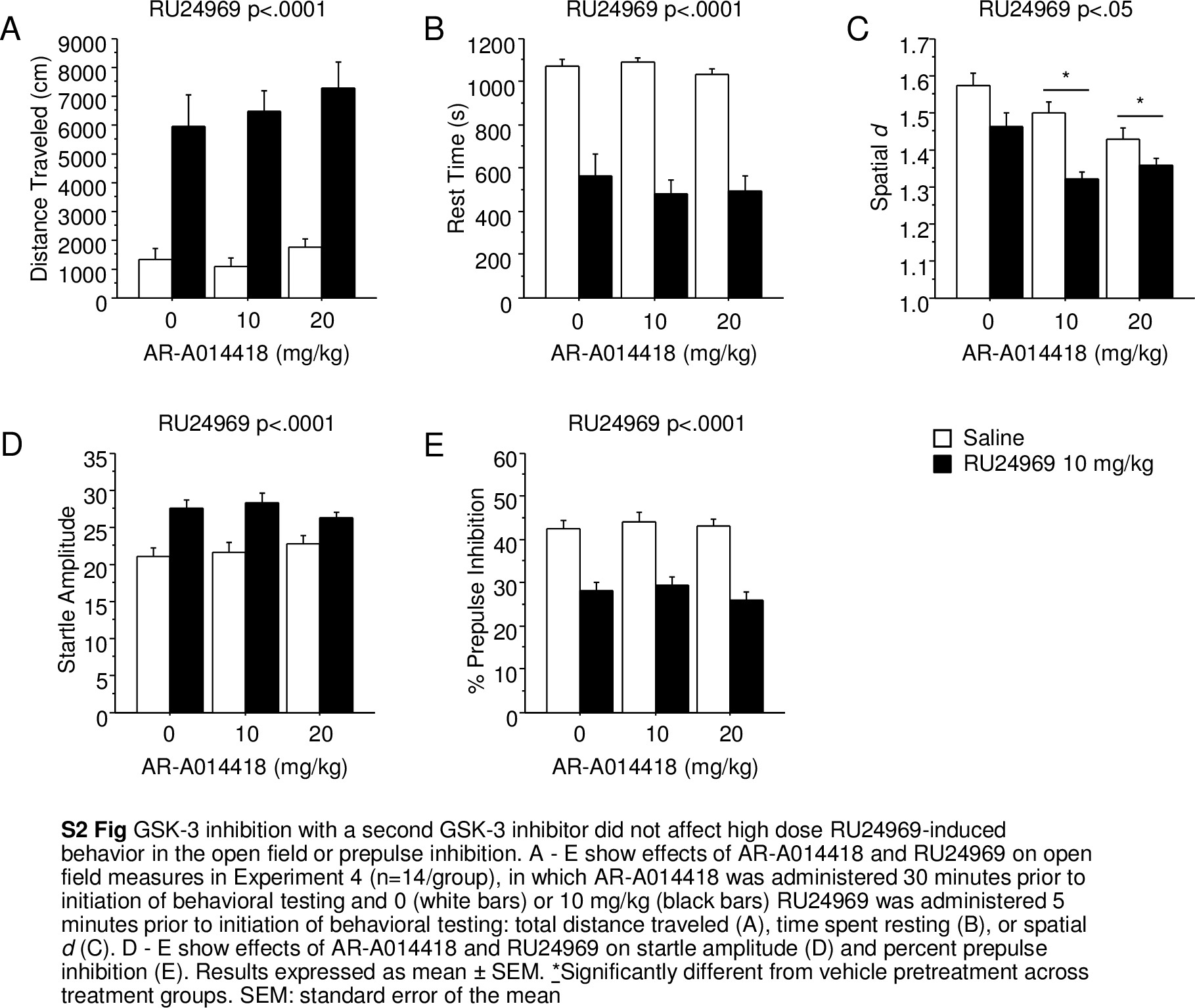

Supplement: S2 Fig — A—E show effects of AR-A014418 and RU24969 on open field measures in Experiment 4 (n = 14/group), in which AR-A014418 was administered 30 minutes prior to initiation of behavioral testing and 0 (white bars) or 10 mg/kg (black bars) RU24969 was administered 5 minutes prior to initiation of behavioral testing: total distance traveled (A), time spent resting (B), or spatial d (C). D—E show effects of AR- A014418 and RU24969 on startle amplitude (D) and percent prepulse inhibition (E). Results expressed as mean ± SEM. *Significantly different from vehicle pretreatment across treatment groups. SEM: standard error of the mean. (TIF) [file pone.0211239.s002.tif]
