# Supplementary material for: Dissecting the roles of β-arrestin2 and GSK-3 signaling in 5-HT1BR-mediated perseverative behavior and prepulse inhibition deficits in mice
Source: PLoS One. 2019 Feb 5;14(2):e0211239. doi: 10.1371/journal.pone.0211239 (PMC6363181; doi:10.1371/journal.pone.0211239)
Supplement: S1 Supplemental Results — Associated with S1 and S2 Figs. (PDF) [file pone.0211239.s003.pdf]

Dissecting the roles of β-arrestin2 and GSK-3 signaling in 5-HT1BR-mediated perseverative behavior and prepulse inhibition deficits in mice

Summer L Thompson and Stephanie C Dulawa

Supplemental Results

Results

No effect of SB216763 on low-dose RU24969-induced changes in behavior

In Experiment 1, 3 mg/kg RU24969 treatment increased distance traveled across pretreatment groups ($F_{(1,72)} = 12.12$; $p<.001$; S1A Fig). SB216763 pretreatment had no effect on distance traveled. RU24969 also reduced time spent resting across pretreatment groups for the low dose ($F_{(1,72)} = 14.55$; $p<.0005$; S1B Fig), whereas SB216763 had no effect on rest time. 3 mg/kg RU24969 had no effect on spatial *d* (S1C Fig).

No effect of AR-A014418 on high-dose RU24969-induced changes in behavior

In the open field, RU24969 treatment increased total distance traveled across pretreatment groups ($F_{(1,78)} = 85.57$; $p<.0001$; S2A Fig). AR-A014418 pretreatment did not interact with RU24969 treatment or have a main effect on distance traveled. RU24969 also reduced time spent resting across pretreatment groups ($F_{(1,78)} = 136.45$; $p<.0001$; S2B Fig), while AR-A014418 had no effect on rest time. RU24969 reduced spatial *d* across pretreatment doses ($F_{(1,75)} = 5.54$; $p<.05$; S2C Fig). AR-A014418 also reduced spatial *d* ($F_{(2,75)} = 3.91$; $p<.05$; S2C Fig) at both the 10 mg/kg and 20 mg/kg doses, but did not interact with RU24969 treatment to affect spatial *d*. Virtually no vertical activity was observed in Experiment 4 (data not shown); thus, statistical analysis was not performed. RU24969 treatment increased startle amplitude overall ($F_{(1,78)} = 20.38$; $p<.0001$; S2D Fig), whereas AR-A014418 had no effect on startle.

RU24969 treatment decreased PPI overall ($F_{(1,78)} = 47.24$; p<.0001; S2E Fig), whereas AR-A014418 had no effect on PPI.

Relationship between saline and RU24969-treated activity levels in the open field within *Arrb2* genotype

None of the genotypes had a significant correlation between distance traveled in the saline and 3 mg/kg RU24969 conditions (WT: r = .27; 95% CI: -.11 to .58; p = .16; HT: r = .16; 95% CI: -.23 to .50; p = .42; KO: r = .30; 95% CI: -.09 to .61; p = .12) or between the saline and 10 mg/kg RU24969 conditions (WT: r = .017; 95% CI: -.35 to .38; p = .93; HT: r = .27; 95% CI: -.11 to .58; p = .16; KO: r = .027; 95% CI: -.35 to .40; p = .89). This lack of relationship was further supported by simple linear regression models assessing RU24969-induced distance traveled based on saline-induced distance traveled. For *Arrb2* WT mice, there was no significant regression between saline and 3 mg/kg RU24969 distance traveled ($F_{(1,27)} = 2.08$; p = .16; r^2 = .07) or between saline and 10 mg/kg RU24969 ($F_{(1,27)} = .01$; p = .93; r^2 = .0003). Likewise for HT mice, there was no significant regression between saline and 3 mg/kg RU24969 ($F_{(1,26)} = .69$; p = .41; r^2 = .03) or 10 mg/kg RU24969 ($F_{(1,27)} = 2.15$; p = .15; r^2 =.07). Similarly for KO mice, there was no significant regression for 3 mg/kg ($F_{(1,25)} = 2.55$; p = .12; r^2 = .09) or 10 mg/kg RU24969 ($F_{(1,26)} = .02$; p = .89; r^2 = .001). Furthermore, the effect size of 10 mg/kg RU24969 treatment on distance traveled was substantially larger in WT mice (*d* = -3.43) than in HT (*d* = -2.54) or KO (*d* = -2.53).

None of the genotypes had a significant correlation between time spent resting in the saline and 3 mg/kg RU24969 conditions (WT: r = .083; 95% CI: -.29 to .44; p = .67; HT: r = .011; 95% CI: -.36 to .38; p = .95; KO: r = .058; 95% CI: -.33 to .43; p = .78) or between the

saline and 10 mg/kg RU24969 conditions (WT: r = .023; 95% CI: -.340 to .380; p = .91; HT: r = .291; 95% CI: -.078 to .589; p = .12; KO: r = .027; 95% CI: -.350 to .396; p = .89). This lack of relationship was further supported by simple linear regression models between saline and RU24969-treated conditions. For WT mice, there was no significant regression between saline and 3 mg/kg RU24969 time spent resting ($F_{(1,27)}$ = .19; p = .67; r^2 = .007) or between saline and 10 mg/kg RU24969 ($F_{(1,27)}$ = .07; p = .79; r^2 = .003). Similarly for HT mice, neither 3 mg/kg ($F_{(1,26)}$ = .003; p = .95; r^2 = .0001) nor 10 mg/kg ($F_{(1,27)}$ = .05; p = .83; r^2 = .002) had significant regressions. Finally, KO mice did not have a significant regression for 3 mg/kg ($F_{(1,25)}$ = .09; p = .77; r^2 = .003) or for 10 mg/kg RU24969 ($F_{(1,26)}$ = .49; p = .49; r^2 = .02). Furthermore, the effect size of 10 mg/kg RU24969 treatment on rest time was larger in the WT mice (*d* = 3.31) than in the HT (*d* = 2.59) or KO mice (*d* = 2.45).
